# A study on the diagnostic value of artificial intelligence combined with a contrast-enhanced ultrasound scoring system in partially cystic thyroid carcinoma

**DOI:** 10.3389/fendo.2025.1514185

**Published:** 2025-07-02

**Authors:** Xiao-Hui Yan, Qian Chen, Yu-Wei Xin, Si-Jie Yuan, Jing-Jing Liu, Hai-Yan Jia, Wen-Ya, Yan-Jing Zhang, Wen-Wen Fan, Yu-Fang Zhao, Liang-Ping, Li-Ping Liu

**Affiliations:** ^1^ Department of Interventional Ultrasound, First Hospital of Shanxi Medical University, Taiyuan, Shanxi, China; ^2^ Department of Urology Surgery, Shanxi Provincial People’s Hospital, Taiyuan, Shanxi, China; ^3^ Department of Ultrasound, Peking University People’s Hospital, Beijing, China; ^4^ Department of Ultrasound, Shanxi Bethune Hospital, Taiyuan, Shanxi, China; ^5^ Department of Ultrasound, First Hospital of Shanxi Medical University, Taiyuan, Shanxi, China; ^6^ Department of Interventional Ultrasound, Fifth Medical Center of Chinese People's Liberation Army (PLA) General Hospital, Beijing, China

**Keywords:** scoring system, ACR TI-RADS, partial cystic thyroid nodules, artificial intelligence (AI), contrast-enhanced ultrasound (CEUS)

## Abstract

**Objectives:**

The aim of this study was to investigate the diagnostic value of the contrast-enhanced ultrasound (CEUS) scoring system, artificial intelligence (AI) and the American College of Radiology Thyroid Imaging and Reporting Data System when used by sonographers of different seniority levels individually and in combination for the diagnosis of partial cystic thyroid nodules (PCTNs).

**Materials and methods:**

A retrospective analysis of conventional ultrasound and CEUS images of enrolled patients was performed, and a CEUS scoring system was established. The sensitivity, specificity, and area under the curve (AUC) of CEUS and AI individually and in combination for diagnosis were compared among sonographers with different seniority levels.

**Results:**

A total of 166 nodules (83 benign and 83 malignant) from 152 patients with PCTNs were analyzed in this study. Nine CEUS features of PCTNs were observed and summarized; eight of these features differed between the two groups (all *p* < 0.05) and were included in the CEUS scoring system. CEUS and AI used by junior and senior physicians effectively diagnosed PCTNs. AI improved the diagnostic efficacy of junior physicians. AI assistance combined with CEUS had the best diagnostic efficacy, with an AUC=0.985 for senior physicians and an AUC=0.967 for junior physicians, with no significant difference (*P*>0.05).

**Conclusions:**

The CEUS scoring system established in this study has high diagnostic value for PCTNs. The use of CEUS and AI can improve the diagnostic accuracy of sonographers and improve the prognosis of PCTN patients.

## Introduction

Partial cystic thyroid nodules (PCTNs) have a high prevalence in the population, accounting for approximately 15% to 53.8% of thyroid nodules detected by ultrasonography (1, 2). Cystic changes within thyroid nodules, especially when the cystic component is prominent, are usually considered characteristic of benign nodules. However, 13% to 26% of thyroid cancers exhibit possible cystic changes (3, 4), and approximately 10% to 28% of PCTNs are malignant (5, 6). Therefore, the 2015 American Thyroid Association guidelines recommend fine needle aspiration biopsy of nodules with suspicious malignant ultrasound features (3). However, a cystic component in thyroid nodules has been confirmed to affect the results of fine needle aspiration biopsy (7). Therefore, it is crucial to find a non-invasive and easily accessible method to determine the benignity and malignancy of PCTNs. There are currently a variety of diagnostic methods for thyroid nodules, including Ultrasound、CEUS、FNAB、AI etc (8)。As a new technological science, artificial intelligence (AI)–ultrasound intelligent auxiliary diagnosis systems have the advantages of speed and high diagnostic accuracy. AI can also improve ultrasound accuracy and significantly limit inter-observer variability (9). In addition, contrast-enhanced ultrasound (CEUS) can provide high-quality auxiliary diagnostic information for the differential diagnosis of thyroid tumors. However, there has been no relevant research on a CEUS scoring system or the diagnostic value of AI for PCTNs. In this study, we not only used the high efficiency and strong diagnostic ability of AI and CEUS to establish an innovative CEUS-based scoring system, but also pioneered the use of AI to improve the recognition of routine ultrasound features of PCTNs and the diagnostic accuracy of junior physicians. AI and CEUS were combined to realize the accurate diagnosis of PCTNs.

## Materials and methods

### Patient data

In this retrospective study, data from 152 patients with PCTNs (166 nodules) and pathological results obtained by surgery or puncture biopsy were collected. The patients were divided into benign and malignant groups according to the pathological results.

The inclusion criteria were as follows: 1) clear surgical pathology and biopsy pathology (Bethesda V and VI for malignant nodules, Bethesda II for benign nodules); 2) clear and complete imaging data from conventional ultrasound and CEUS examinations performed before surgery; and 3) a cystic component greater than 5% of the nodule volume.

The exclusion criteria were as follows: 1) multiple adjacent nodules affecting the acquisition of the tumor region of interest; 2) exposure to high-dose radiation, either inadvertently or as part of medical treatment, or neck surgery; and 3) no normal thyroid gland tissue around the nodules.

### Ultrasonography evaluation

#### Ultrasound images of thyroid nodules were collected using the following devices

A GE LOGIQ E9 Doppler diagnostic instrument with a linear array high-frequency probe and a probe frequency of 6–15 MHz or 3–19 MHz and a Philips IU-22 Doppler diagnostic instrument with a linear array high-frequency probe and a probe frequency of 5–12 MHz or 3–19 MHz.

#### Diagnostic methods of sonographers

A senior physician with experience in thyroid ultrasound and two residents with 3 years of experience in thyroid ultrasound diagnosis analyzed PCTNs the images of the enrolled patients, recorded the characteristics of the thyroid nodules, classified the nodules according to the American College of Radiology Thyroid Imaging and Reporting Data System (ACR TI-RADS) guidelines, and reported the ACR TI-RADS scores. A final consensus was reached in cases of disagreement in diagnostic opinions among junior physicians.

#### CEUS image analysis

The same senior physician analyzed and summarized the following CEUS features of PCTNs according to the CEUS enhancement pattern: (1) intensity of enhancement of the solid component of PCTNs: hypo-, iso-, or hyperenhancement; (2) internal homogeneity: homogeneity or heterogeneity; (3) enhancement ring: absent, incomplete or complete; (4) island-like enhancement: iso- or hyperenhancement in part of the nodule and no enhancement in the remainder, with clear demarcation of the cystic–solid interface; (5) nodule border: ill-defined or well-defined; (6) sparse/no enhancement: stellar contrast only or no contrast agent uptake in the solid part of the nodule, as shown on conventional ultrasound; (7) changes in the size of the noncapsular portion of the nodule on conventional ultrasound: enlargement, maintenance, or reduction; (8) speed of wash-in compared with the surrounding thyroid parenchyma: later, synchronous, or earlier; and (9) speed of washout compared with the surrounding thyroid parenchyma: later, synchronous, or earlier. Only three of these features were evaluated for nodules without enhancement after CEUS: the nodule border, the change in size of the noncapsular portion of the nodule on conventional ultrasound, and sparse/no enhancement.

#### Establishment of a CEUS enhanced model scoring system

Positive CEUS indicators were screened for enhancement differences between benign and malignant PCTNs. Each indicator was assigned a value with reference to the scoring method in the literature (10), and the scores were summed to calculate the CEUS enhancement pattern score of the nodules.

### AI–ultrasound intelligent auxiliary diagnosis system

In this study, the AI-SONIC™ Thyroid Ultrasound Intelligent Assisted Diagnostic System from Demetics Medical Technology was used to analyze routine ultrasound images of PCTNs. Before the study began, the researchers mastered the operation of the AI system. The AI-Sonictm Thyroid Intelligent Assisted Diagnostic System is a new artificial intelligence ultrasound-assisted diagnostic technology, which is a fully automated diagnostic system specialized in ultrasound images and based on deep learning, which can provide reference for the differential diagnosis of benign and malignant thyroid nodules through the analysis of thyroid nodule big data. The system is based on the ACR version of TI-RADS, along with the Kwak version of the classification of a comprehensive Intelligent assessment method. The PCTN images were imported into the system, and the system automatically generated the probability of the nodule being benign or malignant: 0 ~ < 0.4 indicated benign, 0.4 ~ < 0.6 indicated suspicious, and 0.6 ~ < 1 indicated malignant. All the nodules were analyzed 3 times, and the highest value was recorded as the result.

### Statistical analysis

SPSS 25.0 and MedCalc 19.3.1 statistical software were used. The measurement data are expressed as the *mean* ± *standard* deviation or (*median*, *interquartile range*). T tests or *Mann–Whitney U nonparametric* tests were used for comparisons between groups. Count data are expressed as percentages, and the *χ^2^* test was used for comparisons between groups. Using the pathological results as the reference standard, the ACR TI-RADS score, CEUS enhancement pattern score and AI-generated benign/malignant probability value were used to draw the receiver operating characteristic (ROC) curves for the diagnosis of PCTNs by senior and junior physicians and the combination. The optimal diagnostic cut-off value, sensitivity, specificity, accuracy, positive predictive value (PPV), negative predictive value (NPV) and AUC were calculated for each curve. The *McNemar test* was used to analyze the differences in sensitivity and specificity between groups, and the *Z test* was used to compare the differences in the AUCs between groups. *P* < 0.05 was considered statistically significant. AI-assisted sonographer diagnoses and the combination of diagnostic methods were assessed through the establishment of a binary logistic regression model, and the model’s predictions were used to establish an ROC curve.

## Results

### Clinical and pathological results

In this study, 166 nodules from 152 patients who met the inclusion criteria were assessed. The included patients had an age range of 16 to 84 years and an average age of 48.1 ± 13.1 years. A total of 83 (50.0%) nodules from 75 (49.3%) patients were benign, and 83 (50.0%) nodules from 77 (50.7%) patients were malignant. All malignant nodules were confirmed by surgical pathology; 36 (43.4%) benign nodules were confirmed by surgical pathology, and 47 (56.6%) were confirmed by fine needle aspiration biopsy. Seven (4.2%) benign nodules did not show contrast agent uptake on CEUS. The basic characteristics of the patients and nodules are shown in **Table 1**.

**Table 1:** Basic information of patients and PCTNs.

Characteristic	Pathology		*P*
	Malignant(*n*=83)	Benign(*n*=83)	
Age (years)	44.1 ± 13.9	52.1 ± 11.0	0.000
Sex			
Male	22	17	0.405
Female	55	58
Size (cm)	(1.9, 1.6)	(2.2, 2.0)	0.700

### Establishment and diagnostic performance of a CEUS enhancement pattern scoring system for PCTNs

**Figure 1:**
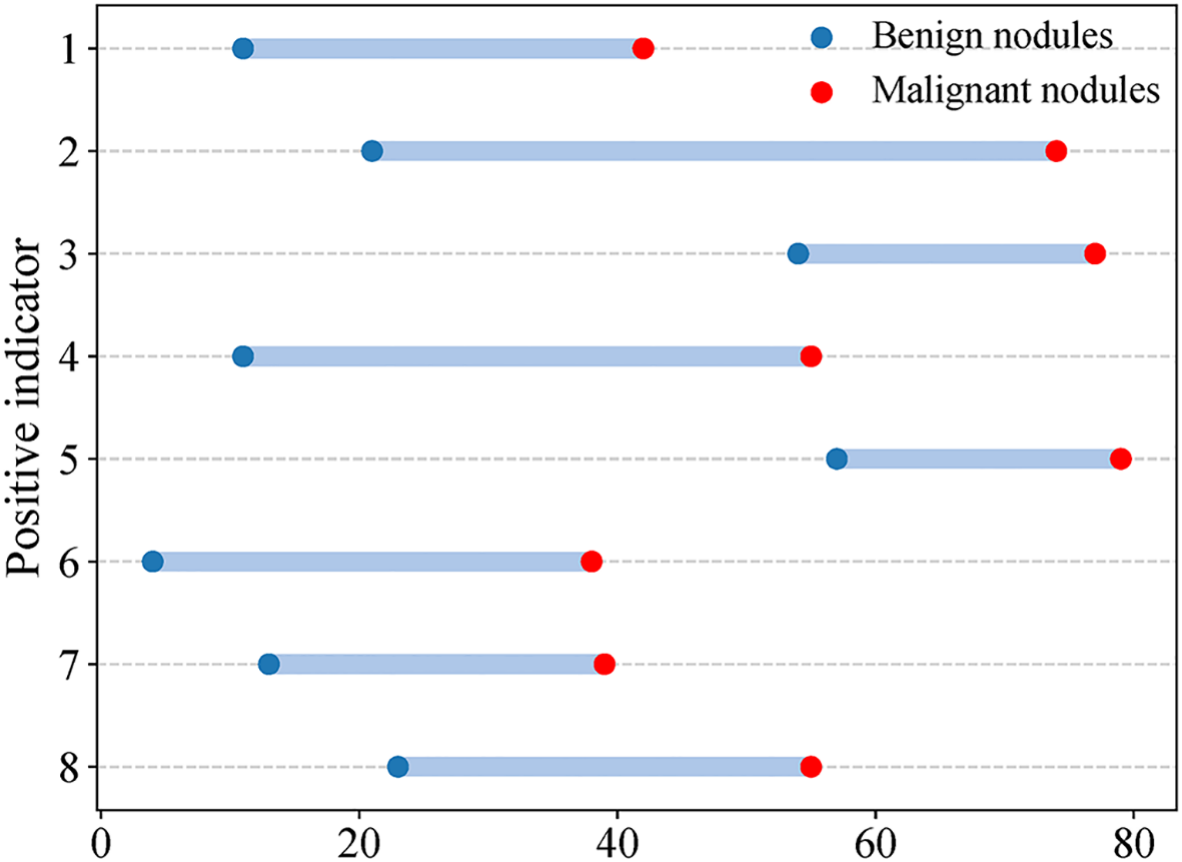
CEUS positive indicators of PCTNs. Indicator 1 Solid part hypoenhancement, indicator 2 No or incomplete enhancement rim around nodule, indicator 3 Non-island enhancement, indicator 4 Nodule boundary ill-defined after enhancement, indicator 5 Non-sparse/no enhancement, indicator 6 Enlarged Solid Part of Nodules on Conventional Ultrasound After Enhancement, indicator 7 wash-in time of solid part of nodules is later than that of surrounding thyroid parenchyma, indicator 8 wash-out time of solid part of nodules is earlier than that of surrounding thyroid parenchyma.

**Figure 2:**
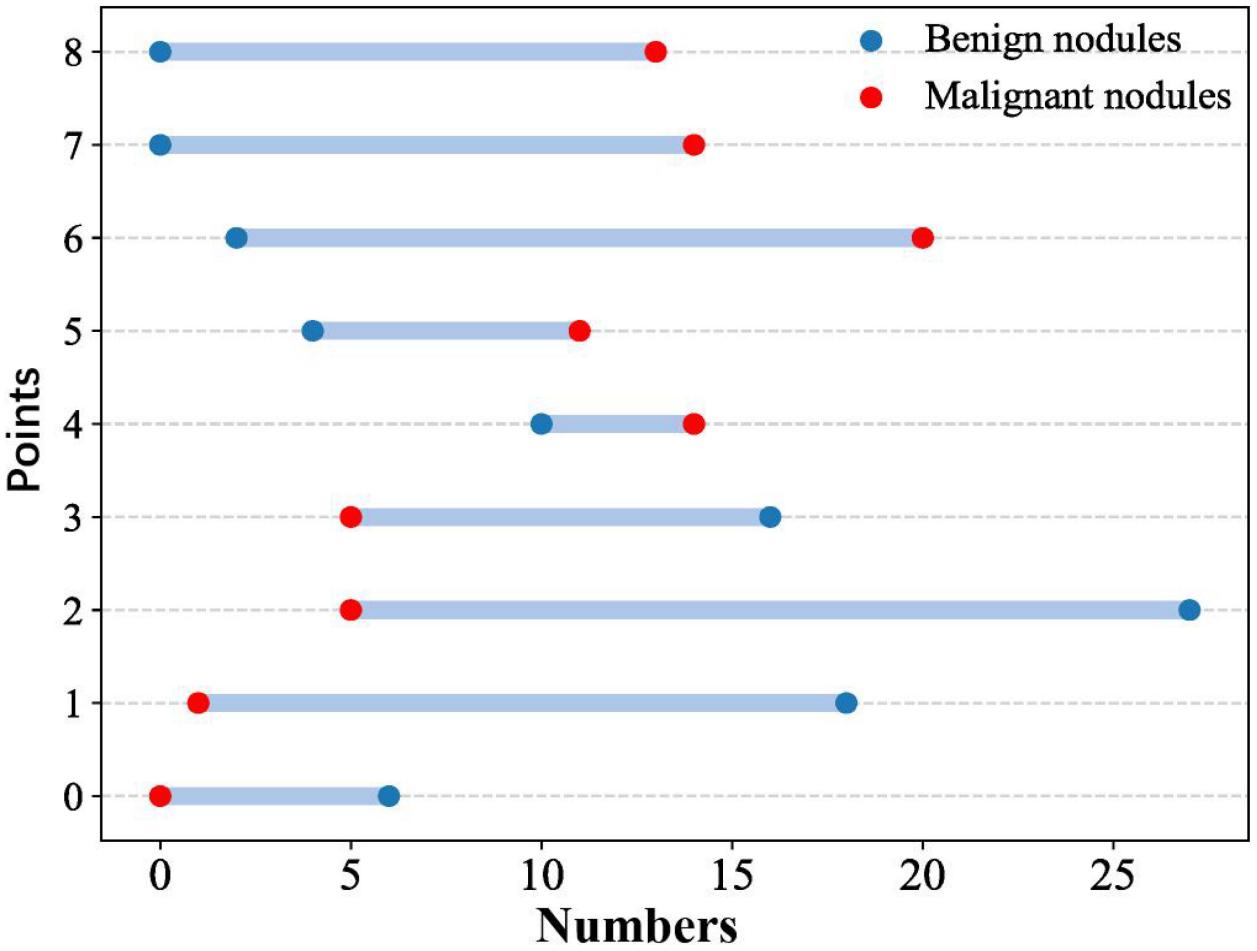
CEUS enhancement pattern score of PCTNs.

**Figure 3:**
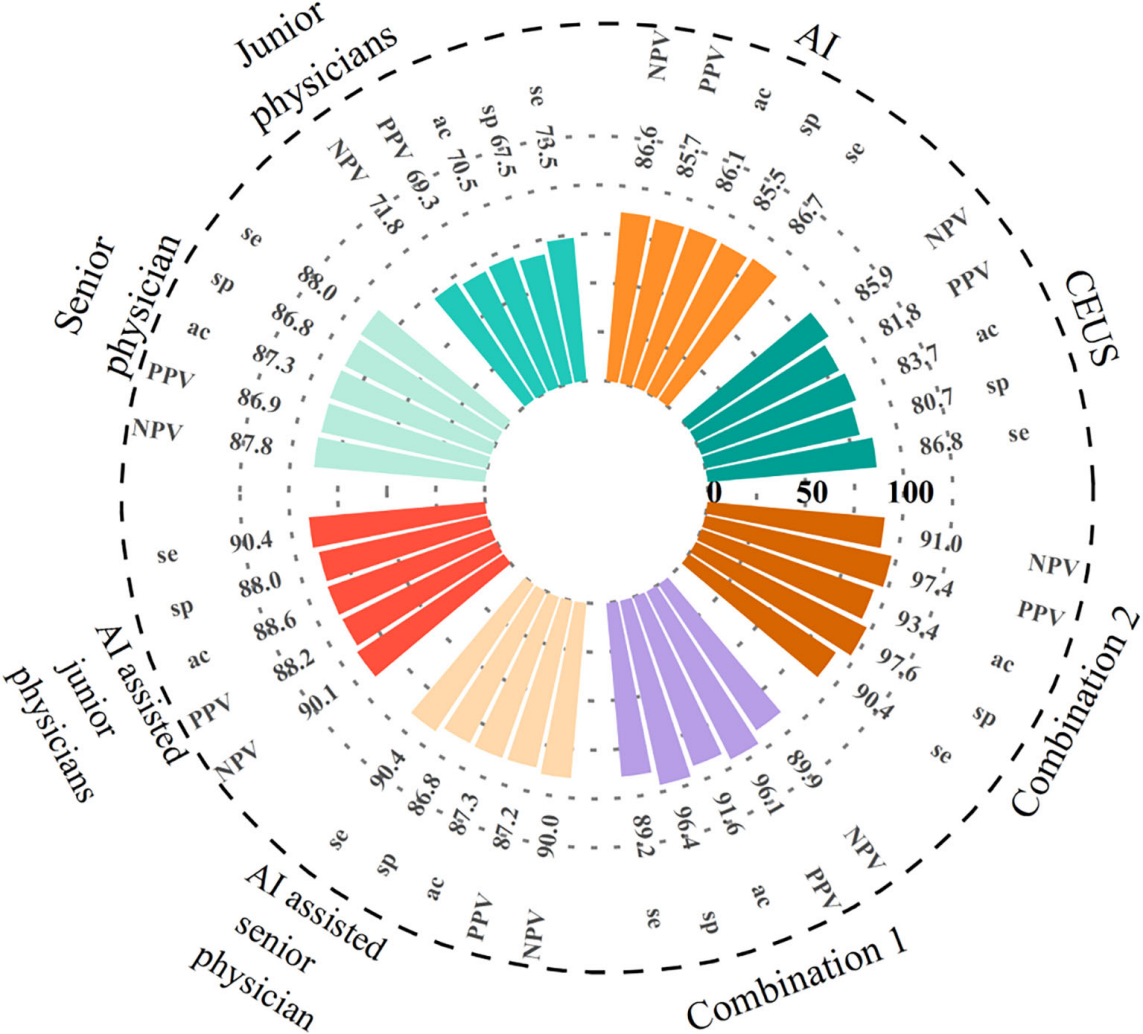
The diagnostic performance of AI, CEUS, different seniority physicians and their combination for PCTNs. Combination of the three methods 1: AI-assisted junior physicians combined with CEUS. Combination of the three methods 2: AI-assisted senior physician combined with CEUS. se, sensitivity; sp, specificity; ac, specificity.

Eight CEUS enhancement patterns were significantly different between the benign and malignant groups (*P* < 0.05); these CEUS enhancement patterns are compared in **Table 2**. According to the characteristics of the CEUS enhancement patterns in the two groups, 8 positive CEUS indicators of PCTNs were defined (**Figure 1**). The CEUS enhancement pattern score of PCTNs is shown in **Figure 2**. The sensitivity, specificity, accuracy, PPV and NPV of the CEUS enhancement pattern score in the diagnosis of PCTNs were 86.8%, 80.7%, 83.7%, 81.8% and 85.9%, respectively, and the AUC was 0.908. The diagnostic performance of the CEUS enhancement pattern score for PCTNs is shown in **Figure 3** and **Table 3**.

**Table 2:** Comparison of CEUS enhancement patterns between benign and malignant PCTNs groups.

CEUS-characteristic	Benign (*n*=76)	Malignant (*n*=83)	*χ^2^*	*Ｐ*
Enhancement ring				
Absent	18 (23.7%)	56 (67.5%)	63.104	0.000
Incomplete	3 (3.9%)	18 (21.7%)		
Complete	55 (72.4%)	9 (10.8%)		
Internal homogeneity				
Homogeneity	36 (47.4%)	30 (36.1%)	2.058	0.151
Heterogeneity	40 (52.6%)	53 (63.9%)		
Island-like enhancement				
Present	22 (28.9%)	6 (7.2%)	12.898	0.000
Absent	54 (71.1%)	77 (92.8%)		
Speed of wash-in				
Later	13 (17.1%)	39 (47.0%)	24.270	0.000
Synchronous	25 (32.9%)	40 (48.2%)		
Earlier	38 (50.0%)	14 (16.9%)		
Speed of wash-out				
Later	15 (19.7%)	7 (8.4%)	20.667	0.000
Synchronous	38 (50%)	21 (25.3%)		
Earlier	23 (30.3%)	55 (66.3%)		
Enhancement intensity				
Hypo	11 (14.5%)	42 (50.6%)	24.852	0.000
Iso	33 (43.4%)	26 (31.3%)		
Hyper	32 (42.1%)	15 (18.1%)		
CEUS-characteristic	Benign (*n*=83)	Malignant (*n*=83)	*χ^2^*	*Ｐ*
Enhancement border				
Ill defined	72 (86.7%)	28 (33.7%)	48.693	0.000
Well defined	11 (13.3%)	55 (66.3%)		
Size change of solid part				
Reduce	23 (27.7%)	6 (7.2%)	40.531	0.000
Monotony	56 (67.5%)	39 (47.0%)		
Enlargement	4 (4.8%)	38 (45.8%)		
Sparse/no enhancement				
Present	26 (31.3%)	4 (4.8%)	19.692	0.000
Absent	57 (68.7%)	79 (95.2%)		

**Table 3:** Diagnostic performance of AI, CEUS, sonographers applied ACR TR and combined methods for PCTNs.

Project	Sensitivity (%)	Specificity (%)	Accuracy (%)	PPV (%)	NPV (%)	AUC
AI	86.7	85.5	86.1	85.7	86.6	0.897
Senior physician	88.0	86.8	87.3	86.9	87.8	0.915
AI Assist senior physician	90.4	86.8	87.3	87.2	90.0	0.960
Junior physicians	73.5	67.5	70.5	69.3	71.8	0.736
AI assisted junior physicians	90.4	88.0	88.6	88.2	90.1	0.939
CEUS	86.8	80.7	83.7	81.8	85.9	0.908
Combination of the three methods 1^*^	89.2	96.4	91.6	96.1	89.9	0.967
Combination of the three methods 2^§^	90.4	97.6	93.4	97.4	91.0	0.985

*Combination of the three methods 1: AI-assisted junior physicians combined with CEUS.

§Combination of the three methods 2: AI-assisted senior physician combined with CEUS.

### Diagnostic performance of sonographers of different seniority levels before and after assistance by AI and the auxiliary system

At a cut-off value of 0.53, the sensitivity, specificity, accuracy, PPV and NPV for AI in the diagnosis of PCTNs were 86.7%, 85.5%, 86.1%, 85.7% and 86.6%, respectively, and the AUC was 0.897. For junior physicians, the application of ACR TI-RADS using an ACR score of 5 to differentiate benign from malignant PCTNs had a sensitivity, specificity, accuracy, PPV, and NPV of 73.5%, 67.5%, 70.5%, 69.3%, and 71.8%, respectively, with an AUC of 0.736. For the senior physician, the application of ACR TI-RADS using an ACR score of 4 to differentiate benign from malignant PCTNs yielded a sensitivity, specificity, accuracy, PPV, and NPV of 88.0%, 86.8%, 87.3%, 86.9%, and 87.8%, respectively, with an AUC of 0.915. The diagnostic performances of AI, junior physicians and the senior physician for PCTNs are shown in **Figure 3** and **Table 3**.

**Figure 4:**
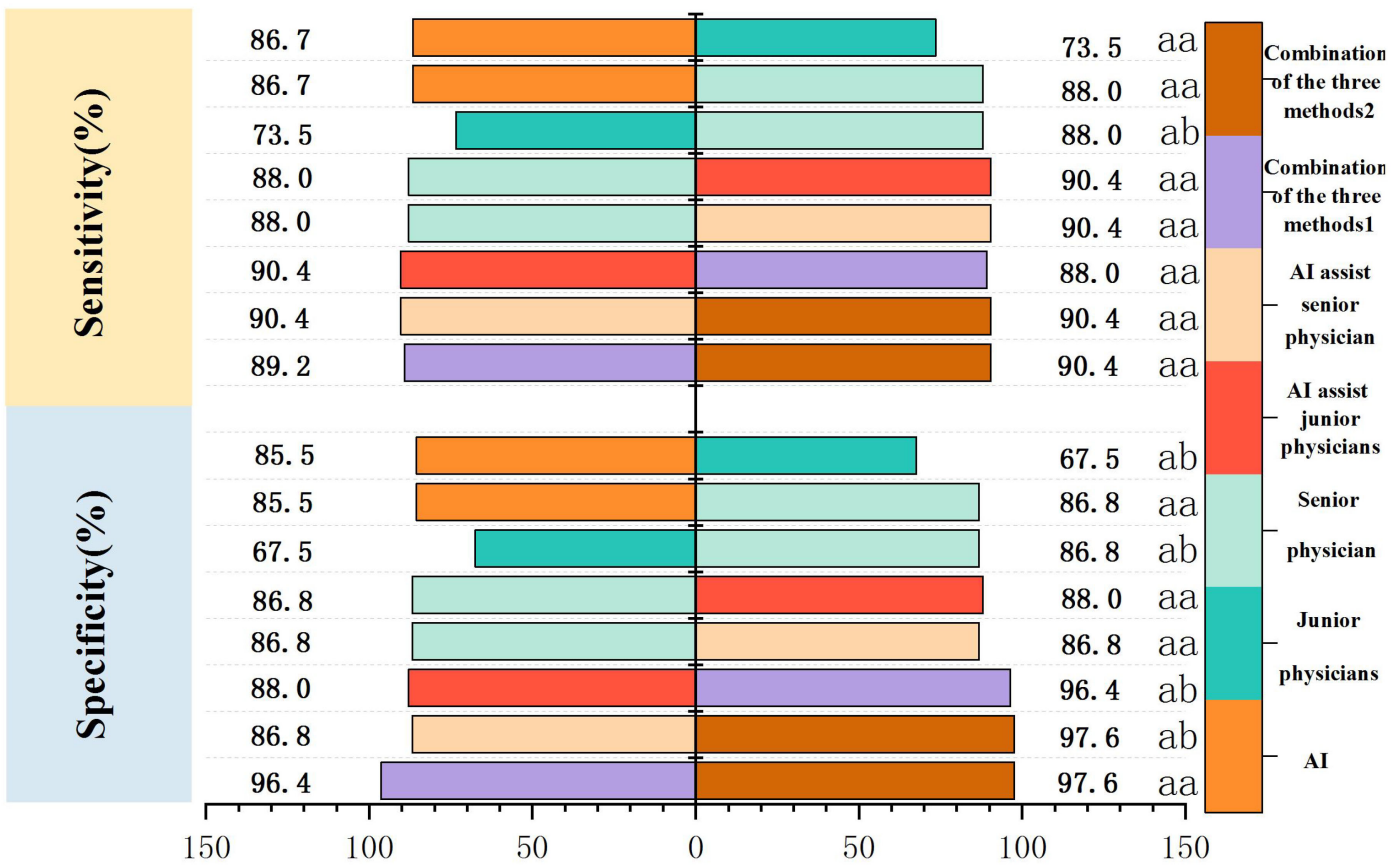
Comparison of sensitivity and specificity of PCTNs diagnosis by AI, different seniority physicians and combined diagnosis. Combination of the three methods 1: AI-assisted junior physicians combined with CEUS. Combination of the three methods 2: AI-assisted senior physician combined with CEUS.

The specificity and AUC of AI in the diagnosis of PCTNs were greater than those of junior physicians (*P* = 0.0046, *P* = 0.0001). The sensitivity, specificity and AUC of AI were comparable to those of the senior physician (*P* = 1.000; *P* = 0.6291; *P* = 0.5793). The sensitivity, specificity and AUC of junior physicians were lower than those of the senior physician (*P* = 0.0290; *P* = 0.0113; *P* = 0.0001). Comparisons of the sensitivity and specificity of PCTN diagnosis by AI and junior and senior physicians are shown in **Figure 4**, and **Table 4**. AUC comparisons are shown in **Table 4**.

**Table 4:** Comparison.

Comparison	Diagnostic methods	Sensitivity		Specificity		AUC		
		Numerical (%)	*P*	Numerical (%)	*P*	Numerical	*Z* statistics	*P*
1	AI	86.7	0.6776	85.5	0.0046	0.897	3.894	0.0001
	Junior physicians	73.5		67.5		0.736		
2	AI	86.7	1.000	85.5	0.6291	0.897	0.554	0.5793
	Senior physician	88.0		86.8		0.915		
3	Junior physicians	73.5	0.0290	67.5	0.0113	0.736	3.854	0.0001
	Senior physician	88.0		86.8		0.915		
4	Senior physician	88.0	1.000	86.8	0.0654	0.915	0.806	0.4200
	AI assisted junior physicians	90.4		88.0		0.939		
5	Senior physician	88.0	0.7905	86.8	0.3877	0.915	2.548	0.0108
	AI Assist Senior physician	90.4		86.8		0.960		
6	AI assisted junior physicians	90.4	0.6250	88.0	0.0391	0.939	2.155	0.0312
	Combination of the three methods1^*^	89.2		96.4		0.967		
7	AI Assist Senior physician	90.4	1.000	86.8	0.0156	0.968	2.204	0.0275
	Combination of the three methods2^§^	90.4		97.6		0.985		
8	Combination of the three methods1^*^	89.2	0.7266	96.4	1.000	0.967	1.450	0.1471
	Combination of the three methods2^§^	90.4		97.6		0.985		

*Combination of the three methods 1: AI-assisted junior physicians combined with CEUS.

§Combination of the three methods 2: AI-assisted senior physician combined with CEUS.

**Figure 5:**
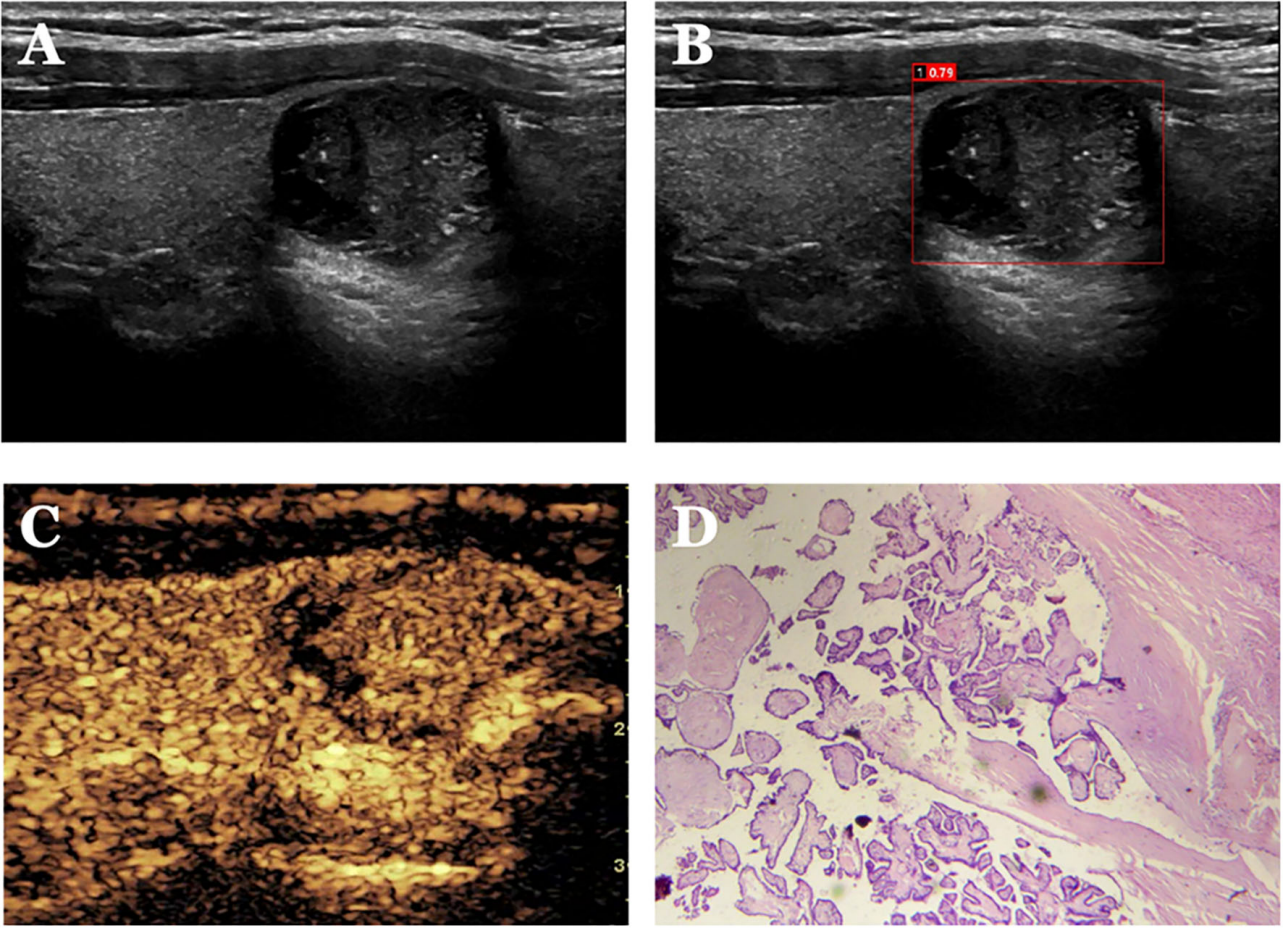
Case 1. The patient is a 34-year-old male. **(A)** shows a cystic solid nodule in the lower pole of the right lobe of the thyroid gland; **(B)** shows a probability value of 0.79 quantified by AI software for benign and malignant, suggesting malignancy; **(C)** shows isoenhancement of the solid part of the nodule by CEUS (identifying dotted strong echogenicity as microcalcifications) with indistinct borders and an incomplete peripheral ring of enhancement, with a CEUS score of 4; **(D)** shows pathological findings of papillary thyroid carcinoma (HE, ×200).

**Figure 6:**
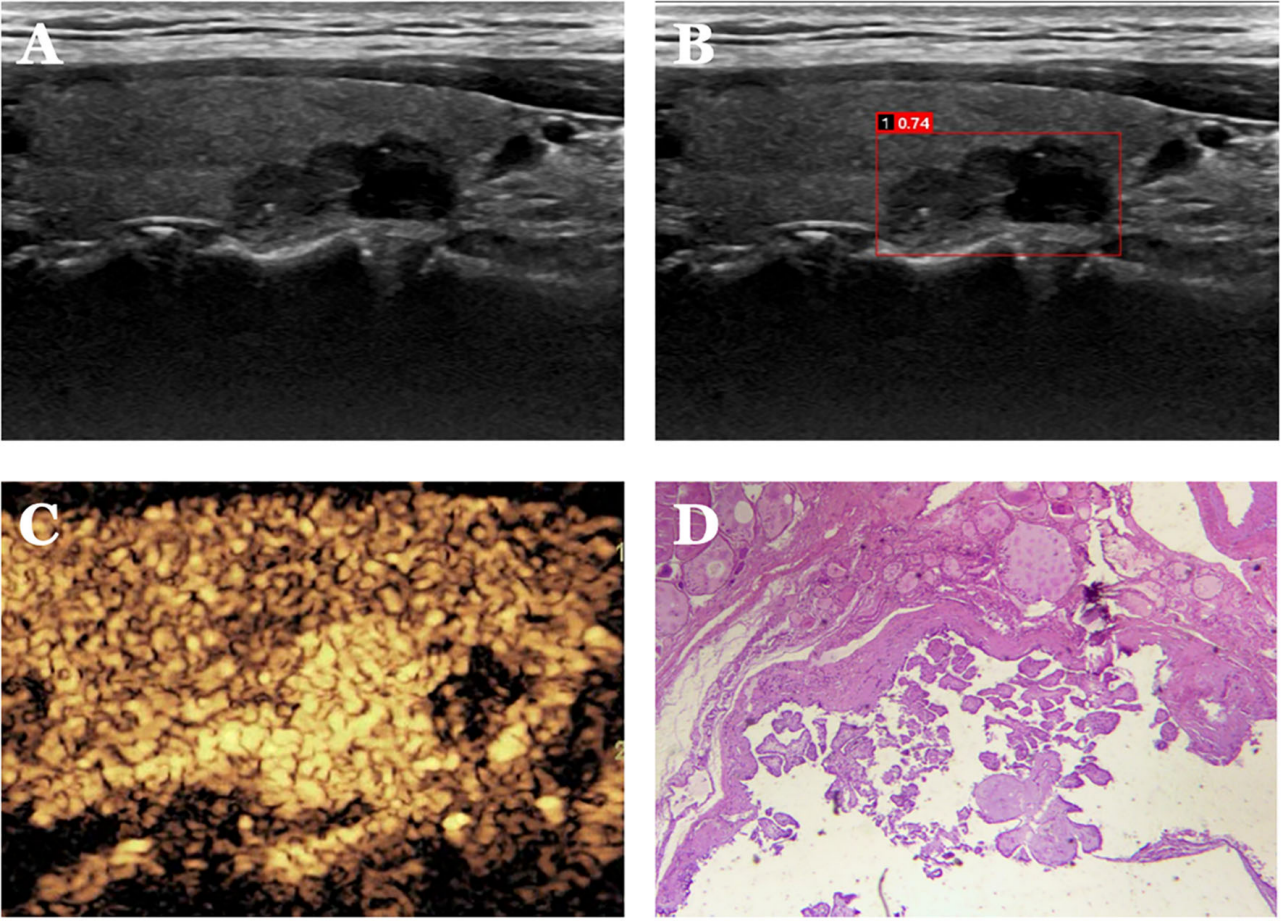
Case 2. The patient is a 37-year-old male. **(A)** Cystic solid nodule in the lower pole of the right lobe of the thyroid gland; **(B)** Probability value of 0.74 quantified by AI software for benign and malignant, suggesting malignancy; **(C)** CEUS showing hyperenhancement of the solid part of the nodule (identifying dotted strong echogenicity as microcalcifications) with indistinct borders and an increase in the extent of the implemented part of the nodule after enhancement, with a CEUS score of 5. **(D)** Pathological findings of papillary thyroid carcinoma (HE, ×200).

**Figure 7:**
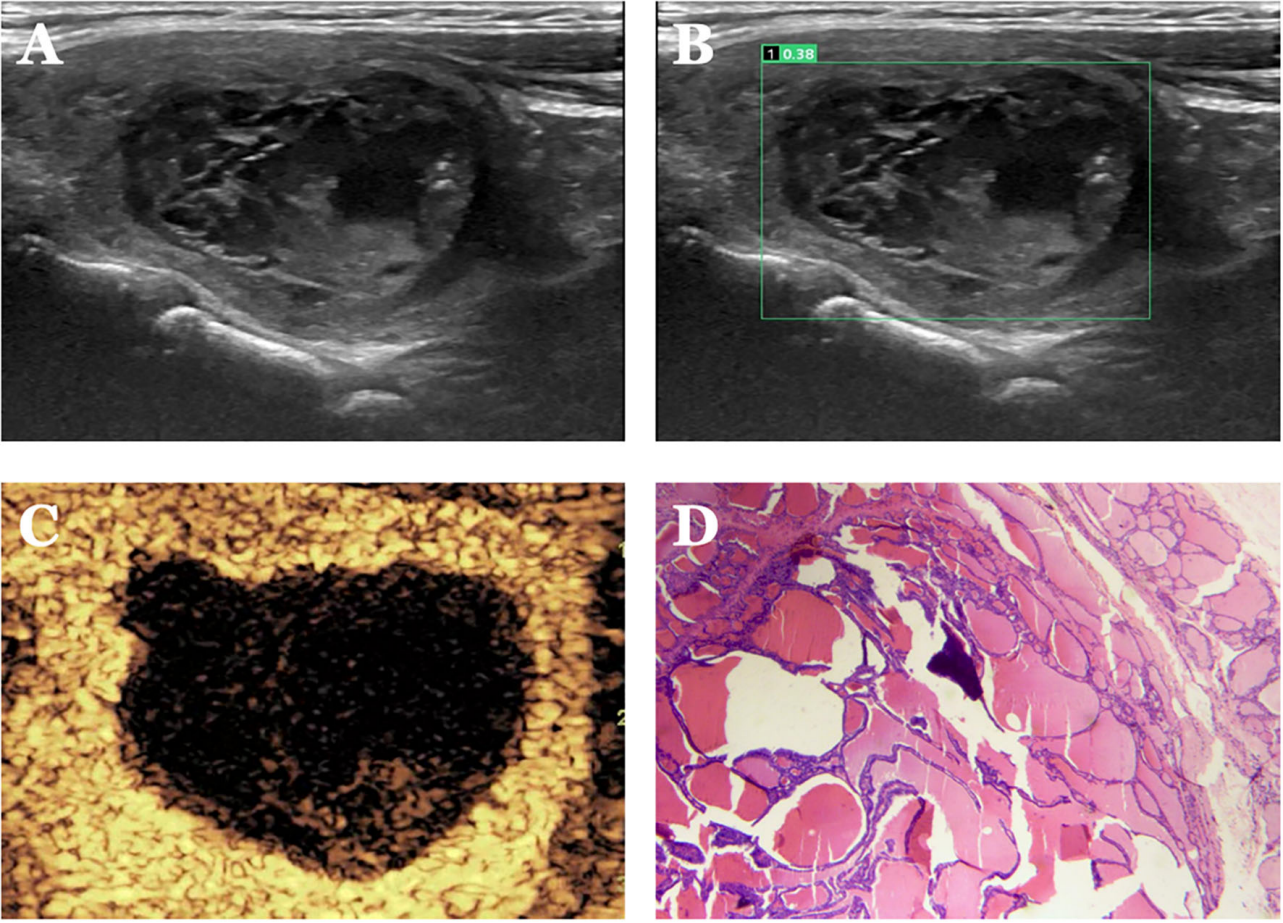
Case 3. The patient is a female, 62 years old. **(A)** Large cystic solid nodule in the left lower middle lobe of the thyroid gland; **(B)** Probability value of 0.38 quantified by AI software for benign and malignant suggests benign. **(C)** CEUS showed isoenhancement of the peripheral cystic wall of the nodule without internal enhancement, and the CEUS score was 2. The diagnosis of benign was accurate after combining AI and CEUS. **(D)** Pathological findings of nodular goiter with hemorrhage and calcification (HE, ×200).

**Figure 8:**
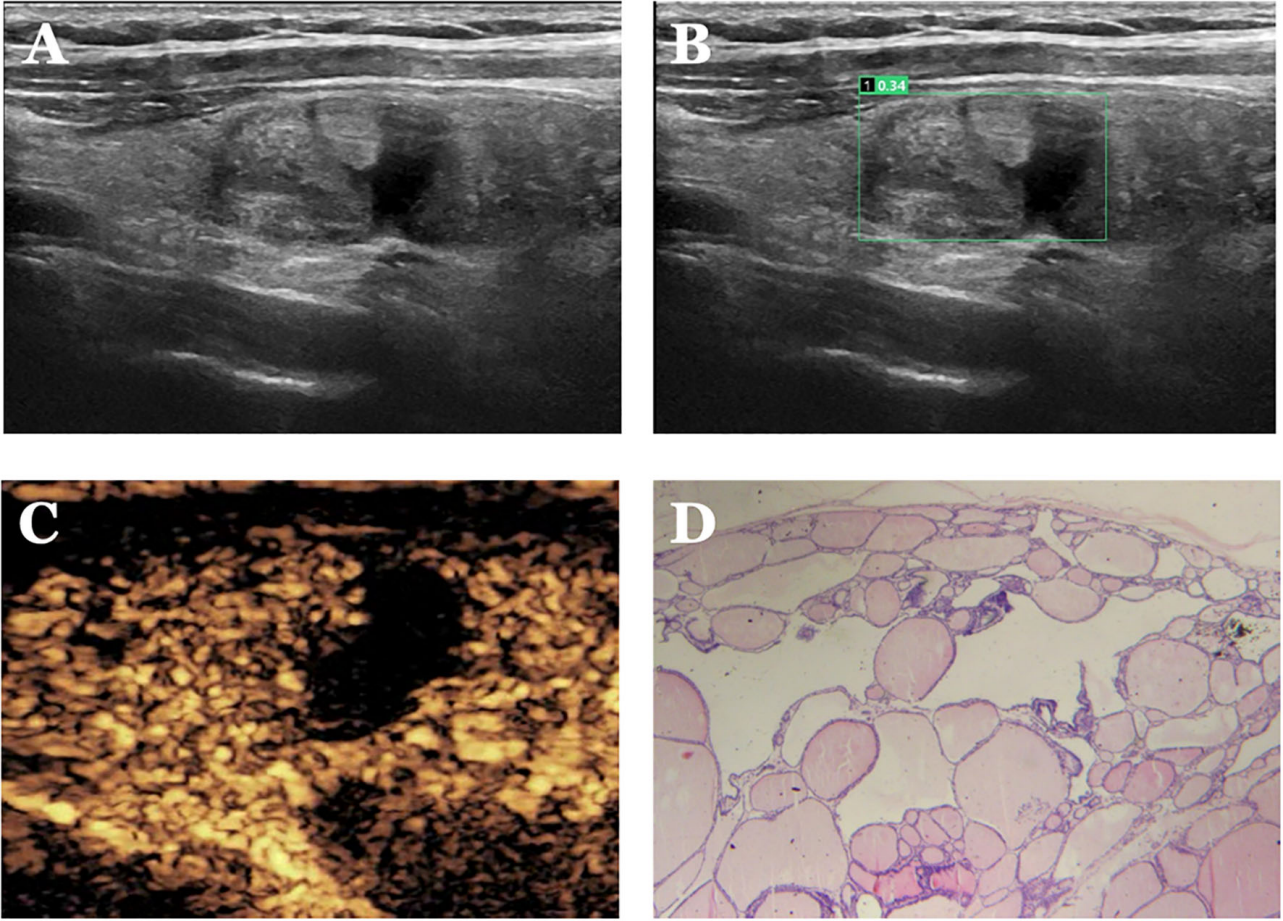
Case 4. The patient is a female, 52 years old. **(A)** shows a cystic nodule in the middle of the right lobe of the thyroid gland; **(B)** shows a probability value of 0.34 quantified by AI software, suggesting benign; **(C)** shows isoenhancement of the solid part of the nodule with a clear cystic-solid interface and “island-like” enhancement, with a CEUS score of 1. **(D)** Pathological findings of thyroid nodular goiter (HE, ×200).

Junior physicians with AI assistance had a sensitivity, specificity, accuracy, PPV, and NPV of 90.4%, 88.0%, 88.6%, 88.2%, and 90.1%, respectively, with an AUC of 0.939 for the diagnosis of PCTNs. Compared with the senior physician, the junior physicians with AI assistance exhibited varying degrees of improvement in each ACR TI-RADS index and comparable diagnostic performance for PCTNs with that of the senior physician, with no significant differences in diagnostic sensitivity, specificity or AUC (*P*=1.000; *P*=0.0654; *P*=0.4200). The diagnostic performance of AI-assisted PCTN diagnosis by junior physicians is shown in **Figure 3** and **Table 3**. A comparison of the sensitivity and specificity of the AI-assisted PCTN diagnosis between junior and senior physicians is shown in **Figure 4** and **Table 4**. A comparison of the AUCs is shown in **Table 4**. **Figures 5**–**8** is referenced below as a clinical reference case.

With AI assistance, the senior physician had a sensitivity, specificity, accuracy, PPV, and NPV of 90.4%, 86.8%, 87.3%, 87.2%, and 90.0%, respectively, with an AUC of 0.960 for the diagnosis of PCTNs. For the senior physician, the AUC for the diagnosis of PCTNs improved after AI assistance (*P*=0.0108). The diagnostic performance of the senior physician with AI assistance is shown in **Figure 3** and **Table 3**. The sensitivity and specificity of the senior physician with AI assistance in diagnosing PCTNs are compared in **Figure 4** and **Table 4**. A comparison of the AUCs is shown in **Table 4**.

### Diagnostic performance of physicians with different levels of seniority assisted by AI combined with CEUS for PCTNs

The sensitivity, specificity, accuracy, PPV and NPV of junior physicians assisted by AI combined with CEUS for the diagnosis of PCTNs were 89.2%, 96.4%, 91.6%, 96.1% and 89.9%, respectively, and the AUC was 0.967. Compared with those of junior physicians with AI assistance alone, the specificity and AUC for PCTN diagnosis were improved for junior physicians with AI assistance combined with CEUS, and the differences were significant (*P* = 0.0391; *p* = 0.0312). The diagnostic performance of junior physicians with AI assistance combined with CEUS for PCTNs is shown in **Figure 3** and **Table 3**. The sensitivity and specificity of junior physicians with AI assistance alone and combined with CEUS for the diagnosis of PCTNs are compared in **Figure 4** and **Table 4**. A comparison of the AUCs is shown in **Table 4**.

The senior physician with AI assistance combined with CEUS had a sensitivity, specificity, accuracy, PPV, and NPV of 90.4%, 97.6%, 93.4%, 97.4%, and 91.0%, respectively, with an AUC of 0.985 for the diagnosis of PCTNs. Compared with those of the senior physician with AI assistance alone, the specificity and AUC of the diagnosis of PCTNs by the senior physician with AI assistance combined with CEUS were improved, and the differences were significant (*P*=0.0156; *P*=0.0275). There was no difference in sensitivity, specificity, or AUC between junior and senior physicians with AI assistance combined with CEUS (*P*=0.7266; *P*=1.000; *P*=0.1471). The diagnostic performance of the senior physician with AI assistance combined with CEUS for PCTNs is shown in **Figure 3** and **Table 3**. The sensitivity and specificity of the senior physician with AI assistance alone and combined with CEUS for the diagnosis of PCTNs are compared in **Figure 4** and **Table 4**. A comparison of the AUCs is shown in **Table 4**.

## Discussion

CEUS qualitative analysis has important value in differentiating benign and malignant thyroid nodules (11), but most studies have focused on solid thyroid nodules. In this study, we not only comprehensively observed and compared 9 CEUS enhancement patterns of PCTNs but also applied a CEUS enhancement pattern scoring method to the differential diagnosis of PCTNs for the first time and achieved good diagnostic performance.

Although the enhancement patterns of benign and malignant PCTNs overlap, the positive CEUS indicators screened in this study are consistent with the enhancement patterns of malignant thyroid nodules reported by most scholars (12–16). Further evaluation of the CEUS score revealed superior diagnostic performance for PCTNs, with an AUC of 0.908, which was comparable to that of AI (AUC=0.897) and the senior physician (AUC=0.915) (both *P*<0.05). These findings indicate that the CEUS score may be a better choice for the diagnosis of PCTNs. It is difficult to compare different studies because there is no uniform standard for enhancement patterns, but the CEUS score has shown good diagnostic performance in similar studies (17–19). However, the following points should be noted: (1) In this study, hypoenhancement of the solid component of the tumor is defined as a “positive indicator”, but the degree of enhancement of the solid component of PCTNs is still controversial, and related studies suggest that PCTNs have a rich blood supply compared with solid thyroid malignant nodules and thus have a greater possibility of showing isoenhancement (20); therefore, when the diagnosis of in single-enhancement mode is difficult, the enhancement characteristics of nodules should be observed from multiple angles and combined with multimodal ultrasound for further diagnosis. (2) In this study, the proportion of benign PCTNs with island-like enhancement was greater than that of malignant nodules (*P* < 0.05), but some studies have shown that the sensitivity of diagnosing benign nodules with island-like enhancement is only 62.5% (21). Therefore, when identifying benign and malignant PCTNs with conventional ultrasound is difficult, island-like enhancement may provide valuable distinguishing information, but clinicians should be aware that benign and malignant nodules can exhibit overlapping enhancement patterns. (3) In this study, the proportion of benign PCTNs with sparse or no enhancement was significantly greater than that of malignant PCTNs; therefore, sparse or no enhancement may provide valuable information for an accurate diagnosis (22).

AI can effectively reduce the variation between diagnosticians and improve the effectiveness of diagnosis using images (17, 23, 24). In this study, an innovative AI-assisted diagnostic system was applied to the differential diagnosis of PCTNs and combined with the CEUS score, which not only compensates for the limitations of subjective interpretation by physicians but also improves the accuracy of PCTN diagnosis by combining tumor microcirculation perfusion information.

The AI-SONIC™ Thyroid Ultrasound Intelligent Auxiliary Diagnosis System from Demetics Medical Technology showed superior diagnostic performance for PCTNs, and the sensitivity and specificity were comparable to those of the senior physician (AUC=0.915), with no statistically significant difference. This finding is similar to those of studies on the diagnostic ability of various computer-aided diagnostic systems for thyroid nodules in China and abroad (25, 26). This finding indicates that the AI-assisted diagnostic system can provide more reliable diagnostic information for the accurate diagnosis of PCTNs. Although PCTNs can be effectively diagnosed by junior physicians (AUC=0.736), all diagnostic indicators of junior physicians were significantly lower than those of AI and senior physicians (both *P*<0.05). Owing to the lack of clinical diagnostic experience, junior physicians have insufficient knowledge of the conventional ultrasound characteristics of PCTNs, and further training of junior physicians to strengthen their clinical diagnostic ability is necessary. When AI was used to assist junior physicians, their diagnostic ability significantly improved and was comparable to that of senior physicians. AI assistance also improved the diagnostic performance of senior physicians for PCTNs. Thus, the AI system can provide relatively objective diagnostic criteria and reduce the difference in diagnostic accuracy between physicians with different levels of experience in different hospitals (27). Finally, after combining AI and CEUS with the ACR TI-RADS classification, the AUCs of junior and senior physicians in diagnosing PCTNs were 0.967 and 0.985, respectively, which were better than those of the other diagnostic methods in this study, indicating that the combination of the three methods can be expected to achieve accurate diagnosis of PCTNs.

In summary, the CEUS enhancement pattern scoring system established in this study, AI and the application of ACR TI-RADS by a senior physician can be used to diagnose PCTNs with a high degree of accuracy. With AI assistance, junior physicians can achieve a diagnostic performance similar to that of senior physicians. AI can also be combined with CEUS to achieve accurate diagnosis of PCTNs. In addition, familiarity with the advantages and disadvantages of the two diagnostic methods of AI and CEUS and proficiency in using these technologies will help sonographers make more rational decisions for patients in their clinical work and reduce their burden.

## Data Availability

The original contributions presented in the study are included in the article/supplementary material. Further inquiries can be directed to the corresponding authors.
